# Ensemble of fine-tuned machine learning models for hysterectomy prediction in pregnant women using magnetic resonance images

**DOI:** 10.1117/1.JMI.12.2.024502

**Published:** 2025-03-18

**Authors:** Vishnu Vardhan Reddy Kanamata Reddy, Michael Villordon, Quyen N. Do, Yin Xi, Matthew A. Lewis, Christina L. Herrera, David Owen, Catherine Y. Spong, Diane M. Twickler, Baowei Fei

**Affiliations:** a The University of Texas at Dallas, Department of Bioengineering, Richardson, Texas, United States; b The University of Texas at Dallas, Center for Imaging and Surgical Innovation, Richardson, Texas, United States; c The University of Texas Southwestern Medical Center, Department of Radiology, Dallas, Texas, United States; d The University of Texas Southwestern Medical Center, Department of Obstetrics and Gynecology, Dallas, Texas, United States; e Parkland Health, Dallas, Texas, United States

**Keywords:** deep learning, radiomics, topographic feature maps, vision transformers, classification, magnetic resonance imaging, placenta accreta spectrum, hysterectomy

## Abstract

**Purpose:**

Identifying pregnant patients at high risk of hysterectomy before giving birth informs clinical management and improves outcomes. We aim to develop machine learning models to predict hysterectomy in pregnant women with placenta accreta spectrum (PAS).

**Approach:**

We developed five machine learning models using information from magnetic resonance images and combined them with topographic maps and radiomic features to predict hysterectomy. The models were trained, optimized, and evaluated on data from 241 patients, in groups of 157, 24, and 60 for training, validation, and testing, respectively.

**Results:**

We assessed the models individually as well as using an ensemble approach. When these models are combined, the ensembled model produced the best performance and achieved an area under the curve of 0.90, a sensitivity of 90.0%, and a specificity of 90.0% for predicting hysterectomy.

**Conclusions:**

Various machine learning models were developed to predict hysterectomy in pregnant women with PAS, which may have potential clinical applications to help improve patient management.

## 1 Introduction

Placenta accreta spectrum (PAS) occurs when the placenta fails to separate from the uterus after delivery.^1,2^ PAS is affecting up to 1 in 272 pregnancies.^3–6^ In cases of PAS, there may be substantial bleeding, which requires immediate removal of the uterus to save the mother’s life. These urgent operations result in significant blood loss and complications.^7–9^ Identification of patients at risk of hysterectomy improves outcomes.^10,11^ Given the rising incidence rate of cesarean delivery and PAS, this is clinically significant.^12^

Convolutional neural networks (CNNs) have performed admirably in the field of computer vision.^13^ Numerous methods based on 2D and 3D CNN have been developed, particularly for the analysis of 3D medical images.^14–17^ 3D-based techniques can naturally learn 3D representations.^18–20^ In the meantime, transformer networks are frequently utilized in both computer vision^21–23^ and natural language processing.^24,25^ More recently, the pure transformer-based computer vision architecture, Vision Transformer (ViT),^22,26^ was able to reach state-of-the-art performance on image classification tasks. CNNs have significant inductive biases and locals that enable them to perform well even with little data. However, these biases may limit their effectiveness when dealing with high-dimensional data that require coverage beyond their typical low receptive field.^23,27^ The layout of a transformer architecture, with its modest inductive biases, can cover a vast region with a high receptive field. On the other hand, these biases might pose limitations when working with small datasets.^22,28,29^

Recently, research has been done on a hybrid network, which combines CNN- and transformer-based architectures, to benefit from both approaches and produce more competitive performances in comparison to traditional approaches.^27–29^ In the field of medical imaging, there are fewer datasets available than in other fields due to ethical concerns,^30,31^ high computational costs,^32^ expensive annotation,^32^ and significant class-imbalance issues.^33^ However, recent studies showed that combining 3D CNNs, 2D CNNs, and transformers leads to synergic effects, with the resulting network achieving state-of-the-art results for 3D medical image classification even with a smaller dataset.^34^

Radiomics, a novel machine learning approach, endeavors to quantify phenotypic traits on medical images through automated algorithms, facilitating the extraction of a high-dimensional set of characteristics from clinical data for quantitative analysis of radiologic data.^35,36^ In earlier research, it was discovered that placenta and uterus volumes segmented using deep learning and expert segmentation shared roughly 40% of the same radiomic characteristics.^37–39^ Using a combination of radiomics and deep learning, a different group was able to predict placental invasion on T2-weighted magnetic resonance imaging (MRI) with 0.94 accuracy.^40^ In a study employing radiomics to predict hysterectomy due to PAS, the best model had an area under the curve (AUC) of 0.80 for a cohort of 62 individuals.^41,42^ A study employing MRI to predict hysterectomy and PAS in pregnant women achieved a classification accuracy of 0.92 and 0.88, respectively, using radiomic characteristics derived from the placenta and uterine in 241 pregnant women’s magnetic resonance (MR) images.^43^ Deep learning methods using the different modalities of data, such as 3D MRI, 2D topographic feature maps, and radiomics achieved promising results in predicting PAS in pregnant women severe enough to warrant a hysterectomy after infant delivery.^44^ Architectures, such as CascadeNet, were used to process the multimodal data for hysterectomy prediction and achieved an AUC of 0.878, accuracy of 83.3%, sensitivity of 85.0%, and specificity of 82.5%.^44^

Building upon the promising results of combining deep learning and radiomics for hysterectomy prediction, this study proposes a novel multilevel ensemble approach. Through the use of ensemble learning, data from various classification models are combined into a single, superior classifier to produce improved prediction performance.^45^ Expert-level predictions are provided for challenging medical image classification through the ensemble of neural networks.^46,47^ We leveraged the strengths of 3D CNNs, transformers, and statistical machine learning models by processing 3D MRI scans, topographic feature maps, and radiomics through five individual models. By combining the predictions from these models using a hard voting mechanism, we achieved superior performance in predicting the need for hysterectomy in pregnant patients with prenatal concern for PAS. This approach has the potential to improve clinical decision-making and patient outcomes by providing expert-level predictions.

## 2 Methods

### 2.1 MRI Data

The data used in our study consist of 241 T2-weighted MRI data (1.5T) from 241 pregnant women, grouped by their clinical outcome: those who eventually had a hysterectomy (88) and those who did not (153). The size of the axial images was 256 × 256 pixels, with the exception of three patients where the in-plane sizes were zero-padded to 256 × 256 pixels. The slice thickness was 7.0 mm, with the total number of slices per patient ranging from 28 to 62. The in-plane resolution of the axial images ranged from 1.055 × 1.055 mm^2^ to 1.953 × 1.953 mm^2^ across all patients. The number of slices varies because the size of the uteruses varies among different subjects. Following our previous work by Dormer et al.,^44^ who used the same dataset, the through-plane variability was addressed during preprocessing using an isotropic voxel size. This approach has been shown to maintain acceptable quality while enabling consistent 3D analysis across varying fields of view.

The 241 patients were split into groups of *N* = 157 for training, *N* = 24 for validation, and *N* = 60 for testing. The training data consist of 56 hysterectomy cases and 101 no-hysterectomy cases. The validation data consist of 12 hysterectomy cases and 12 no-hysterectomy cases, and finally, the test data consist of 20 hysterectomy cases and 40 no-hysterectomy cases.

### 2.2 Preprocessing

**Fig. 1:**
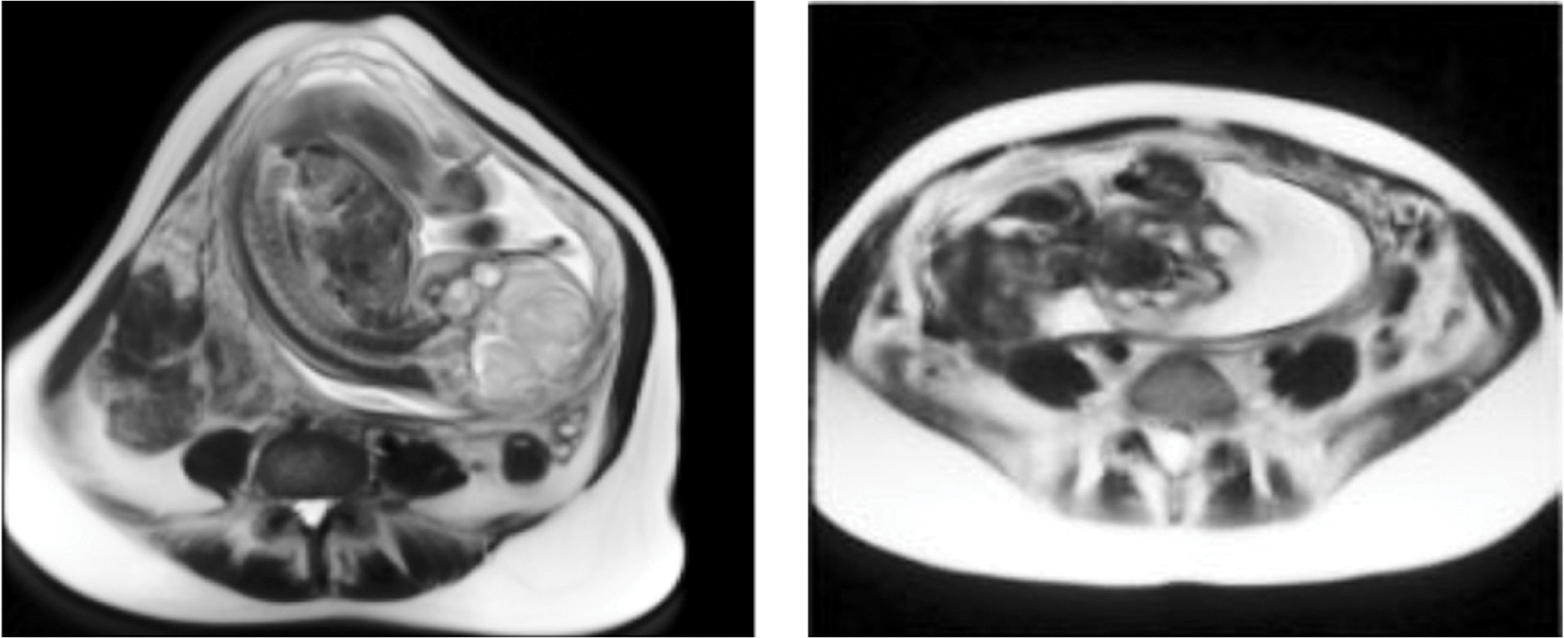
3D MRI slices of the placenta.

The 3D MRI volumes were resized to 192 × 192 × 25 voxels using linear interpolation. Sample 3D MRI slices of the placenta are shown in Fig. 1. The training dataset had an imbalanced number of patients, so an additional technique was used to augment the data. This involved either cropping or padding the original MRI in the in-plane direction by 15% before resizing using linear interpolation to 192 × 192 × 25 voxels. All 56 hysterectomy patients in the training dataset were augmented using both methods, resulting in a total of 168 volumes. For 101 no-hysterectomy cases, those with resolutions below 1.3 × 1.3 mm^2^ were augmented using the cropping method, whereas those with higher resolutions were augmented using the padding method. This doubled the number of normal patients in the training group to 202 and brought the total training dataset to 370 patients. This improved the ratio of hysterectomy to normal patients from roughly 1:2 to 4:5.^44^

### 2.3 Radiomic Features

A radiologist’s manual segmentations of the uterus and placenta were obtained and used as two individual masks to extract the radiomic features. In total, 214 radiomic features were extracted using PyRadiomics, including shape, gray level co-occurrence, gray level run length, and first-order statistics. A total of 107 features were extracted from each of the placenta and uterus regions.^44^

### 2.4 Topographic Feature Map

**Fig. 2:**
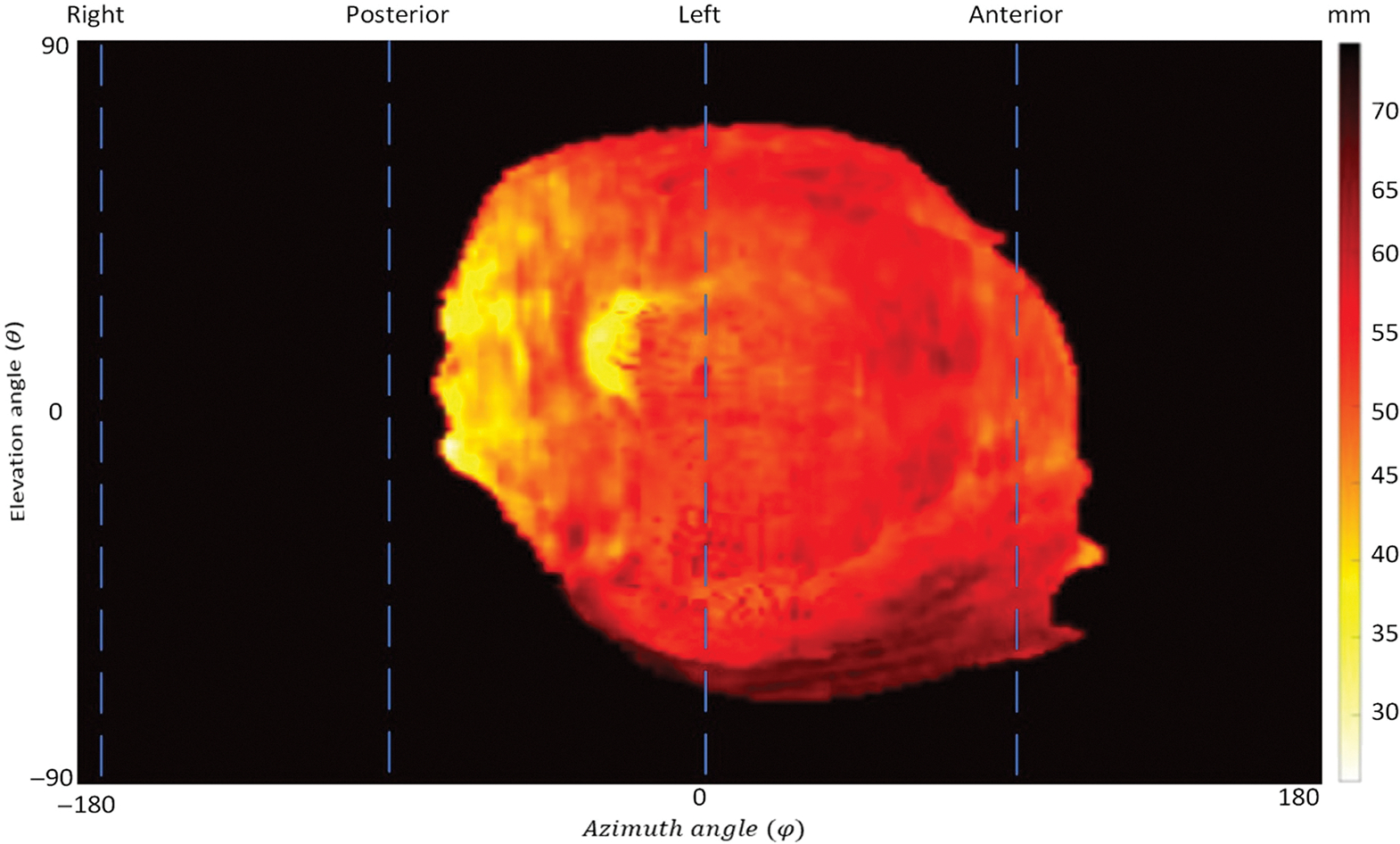
Topographic feature map of the placenta. The superior and inferior sides are depicted, respectively, in the top and bottom halves of the topography map.

To gain information about the placenta’s texture and surrounding areas, novel topographic feature maps were made.^48^ We used the distance between surface points and point of view as the displayed feature. Figure 2 shows a sample topographic map. The same topography-based scanning and mapping method was employed to extract and map various features, such as distance, placenta thickness, surface intensity, local mean of intensities, and local standard deviation of intensities.^48^ The patch size for the patch-based extraction was 11 × 11 × 11 voxels.

### 2.5 Major Configurations of the Models

To comprehensively assess the potential of machine learning for predicting hysterectomy in pregnant patients, we developed and compared five distinct model configurations. The first method utilized traditional statistical machine learning on radiomic features extracted from MRI data, establishing a baseline performance benchmark. Next, we fine-tuned the CascadeNet^44^ model with additional improvements. In a previous study,^44^ it was mentioned that their method can be improved with additional fine tuning. For the third method, we explored the potential of transformer-based architectures by replacing the 2D CNN component of CascadeNet with a ViT for processing topographic feature maps derived from MRI data. To address potential data biases and enhance generalizability, we implemented stratified cross-validation on both fine-tuned CascadeNet model and CascadeNet model with ViT. This exploration of various machine learning approaches and data modalities allowed for a systematic evaluation of their effectiveness and culminated in the development of an ensemble model for predicting the need for a hysterectomy.

#### 2.5.1 Model 1—machine learning classifier based on radiomics

In this method, we performed the classification task using 15 machine learning algorithms. The scikit-learn^49^ package in Python was used to implement all machine learning methods with default settings. In addition, we used a machine learning classifier (LightGBMClassifier^50^) implemented utilizing its respective Python package with its default parameters. For this method, we utilized radiomics features extracted from a combined dataset of 394 MR images (370 images from 157 patients in the training set and 24 images from 24 patients in the validation set) to fit all 16 classifiers. There were no patients in the training and testing groups who were the same. Their predictive performance was assessed on the testing set through accuracy, sensitivity, specificity, and ROC-AUC analysis.

After extensive training, we chose the LightGBM classifier as model 1 because it was the only machine learning classifier with high accuracy and AUC. Training only on radiomics allowed the model to have a different understanding of the data than the other ones, allowing for a unique generalization compared with all the other models.

#### 2.5.2 Model 2—modified CascadeNet

**Fig. 3:**
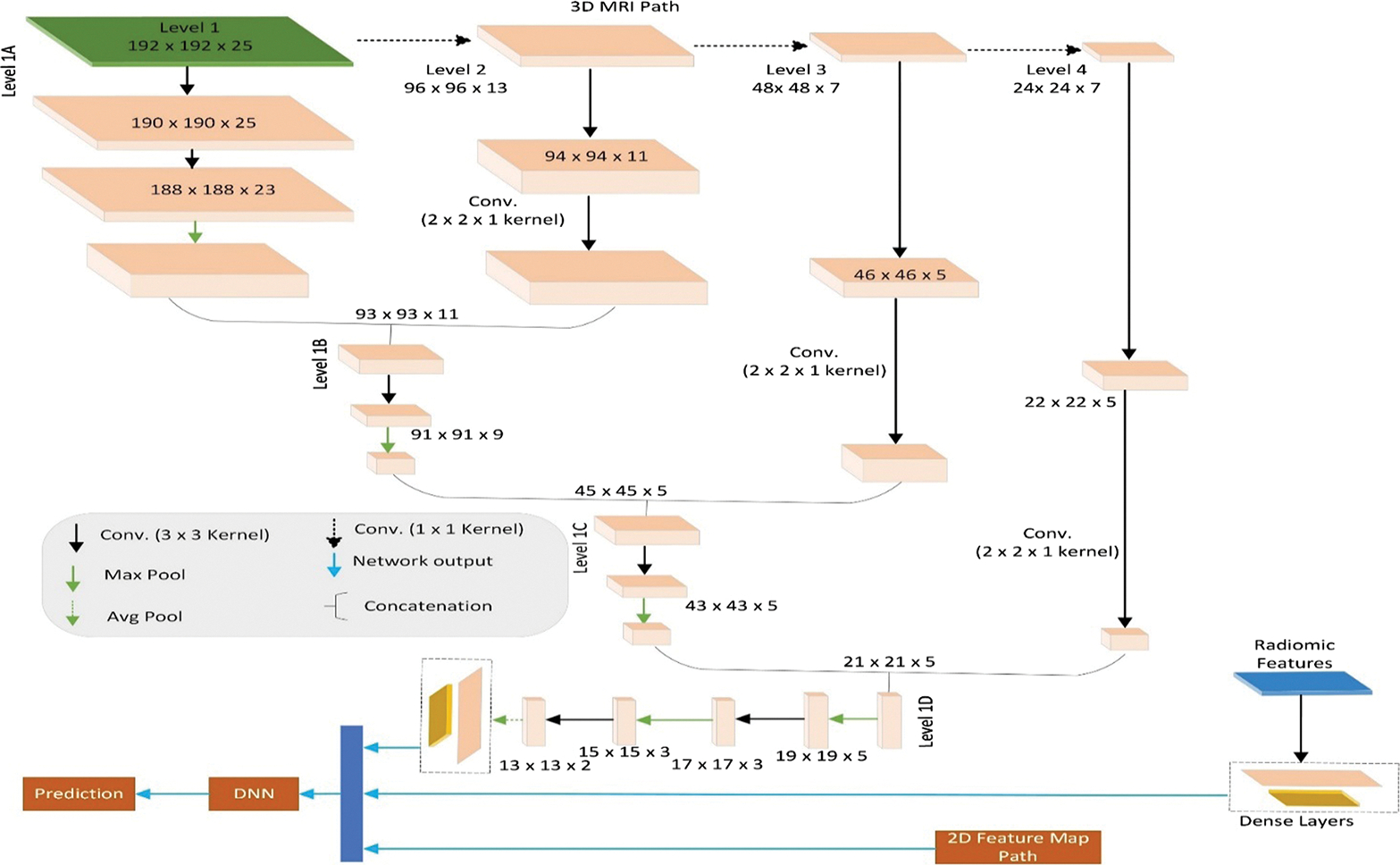
Illustration of modified three-path version of CascadeNet.

**Fig. 4:**
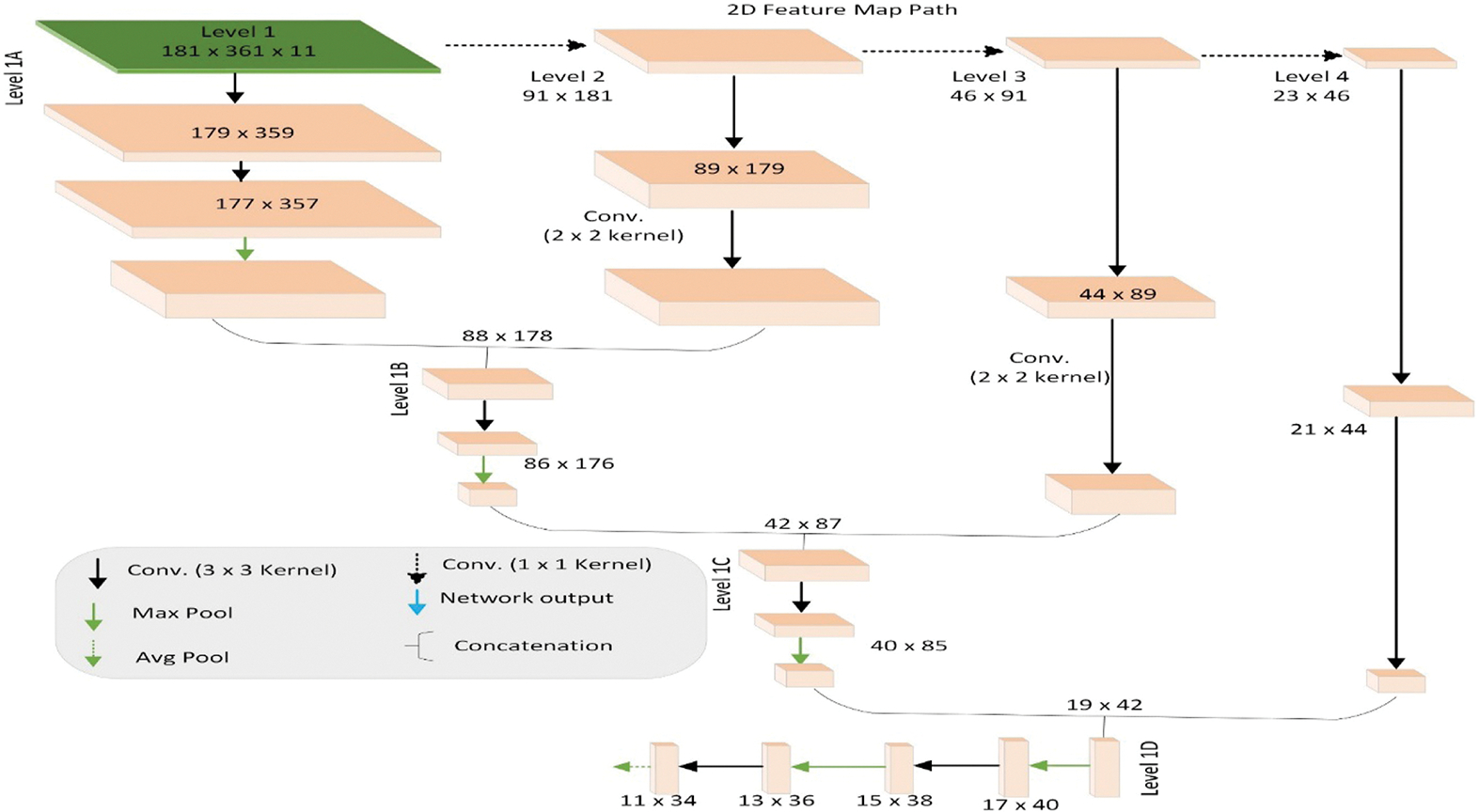
Illustration of 2D topographic feature map processing component of CascadeNet.

The modified CascadeNet architecture is illustrated in Fig. 3, which shows three parallel processing paths: (1) the MRI path utilizing 3D convolutions for volumetric analysis of 3D MRI data, (2) the radiomics path consisting of dense layers for processing radiomic features, and (3) the topographic feature map path employing 2D convolutions, detailed in Fig. 4. Our key innovation lies in the feature fusion mechanism: we introduced additional deep neural network (DNN) layers after the concatenation of features from all three paths. These novel DNN layers were specifically designed to learn complex interactions among different modalities (MRI, radiomics, and topographic features) and extract higher-order patterns from their combined representations, thereby enhancing the model’s classification capabilities. This multimodal feature integration approach represents a significant advancement over the original CascadeNet architecture.^44^

The DNN consists of a multilayer perceptron (MLP) with three layers each with 2000 features followed by the SeLU^51^ activation layer and Lecun normal kernel initialization^51^ followed by alpha dropout^51^ of 0.25. Finally, the prediction layer was a dense layer with two output features followed by a softmax activation function to classify the multimodal input. This modified CascadeNet improved the accuracy of test data by 2% compared with the previous CascadeNet^44^ architecture.

#### 2.5.3 Model 3—modified CascadeNet using cross-validation

To enhance model generalization and address potential data biases, we implemented an alternative training strategy using a stratified sevenfold cross-validation technique^52^ for model selection. In this approach, we combined our training and validation datasets and split them into seven folds while maintaining the class distribution in each fold. The modified CascadeNet was then trained seven times. This process yielded seven different models; each was evaluated on its respective validation fold. The model that achieved the highest performance on its validation fold was selected as our final model and was subsequently evaluated on the independent test set.

#### 2.5.4 Model 4—replacing the 2D CNN component of the CascadeNet with ViT

We also developed an alternative version of our modified CascadeNet where we integrated ViT architecture specifically for processing the 2D topographic feature maps. In this configuration, although the 3D MRI path (with 3D convolutions) and the radiomics path remain unchanged as shown in Fig. 3, we replaced the 2D CNN component with a ViT architecture. The output features from the ViT are concatenated with the features from the MRI and radiomics paths and then processed through our novel DNN layers before final classification. This architectural variation was explored with two objectives: (1) to investigate whether transformer-based feature extraction could capture different patterns in the topographic features compared with traditional 2D CNNs and (2) to evaluate if the self-attention mechanism of ViT could provide complementary information when integrated with features from other modalities. However, this replacement with the ViT network only achieved comparable performance with the modified CascadeNet.

The ViT consists of four transformer layers with a patch size of 6, a hidden size of 128 units, and two heads. The MLP block in the ViT consists of two dense layers of 128 units each followed by a dropout layer of 0.15 and GeLU^53^ activation. The final logit layer consisted of a dense layer of 150 units with ReLU^54^ activation, and the weights were initialized to 0.

#### 2.5.5 Model 5—replacing 2D CNN component of CascadeNet with ViT with cross-validation

The next model we evaluated consisted of the same architecture as model 4 but with stratified cross-validation. We used this technique for model selection by combining both training and validation datasets and training the model for seven folds. As we obtained seven estimates of the model’s performance on its respective validation data, we chose the model with the highest performance out of seven estimates.

### 2.6 Implementation

Each network was trained using the RMSProp optimizer in TensorFlow, with an initial learning rate of 0.00001, adjusted through exponential decay. Empirical results demonstrated that RMSProp achieved faster convergence compared with other optimizers, including Adam and SGD. In addition, RMSProp’s adaptive learning rate was advantageous given the data characteristics, aligning with findings reported by Dormer et al.^44^ Binary cross entropy was used as the loss function, with a weighting factor of 1:1.15 (no hysterectomy: hysterectomy) to account for the imbalanced classes. During training, the dataset is shuffled after every epoch. Due to the nonsymmetric and complex nature of the data, as also noted by Dormer et al.,^44^ who used the same dataset, additional augmentation technique was not employed. The networks were built on a CentOS 7 system with TensorFlow version 2.4 running in Docker. The training was conducted on an NVIDIA A6000 GPU. The evaluation metric used was patient-level accuracy. Once the ideal model for each method was found, these models were combined and evaluated on the reserved testing dataset.

### 2.7 Evaluation Metrics

We use accuracy, sensitivity, and specificity to evaluate the performance of the prediction model. Accuracy is defined as

(1) $$\text{Accuracy}=\frac{\left(TP+TN\right)}{\left(TP+TN+FP+FN\right)},$$

where TP is true positive, TN is true negative, FP is false positive, and FN is false negative. Specificity is the proportion of true negatives that the model correctly predicts, whereas sensitivity is the fraction of true positives that the model correctly predicts.


(2) $$\text{Sensitivity}=\frac{TP}{\left(TP+FN\right)},$$

(3) $$\text{Specificity}=\frac{TN}{\left(TN+FP\right)}.$$

The area under the receiver operating characteristic curve using the probabilistic prediction was also used to evaluate the overall performance of the prediction model.

## 3 Results

### 3.1 Comparison Study Results

The results from radiomics test data when evaluated with 16 machine learning models are shown in Table 1. In our evaluation, we found that the LightGBM classifier outperformed every other model in terms of accuracy and ROC-AUC. Out of the 16 machine learning models, there were 10 models that had at least 70% accuracy and ROC-AUC.

We chose the LightGBM model and the four modified three-path CascadeNet models and compared each model with the CascadeNet.^44^ The quantitative performance is presented in Table 2. Our developed models outperformed the CascadeNet model in 50% of testing (bolded). We have also shown that the CascadeNet can be improved with additional architectural changes (model 3), fine-tuning, and cross-validation. However, when combining all five model’s binary predictions through a majority voting scheme, the ensemble outperformed all its individual models’ performances, giving state-of-the-art results.

In addition, the transformer-based architectures, such as ViT, had lower performance metrics than the modified CascadeNet model, which is of pure CNNs when trained on a small medical image dataset. Although each network achieved comparable performance with CascadeNet,^44^ the sensitivity and specificity metrics show that these networks were able to uncover patterns that were able to confirm the hysterectomy in the event of a positive result. Combining these five models (ensemble) boosted metrics such as accuracy and AUC, thereby achieving robust performance.

The LightGBM classifier (model 1), which was trained only on radiomics data, has achieved high specificity, low sensitivity, and comparable accuracy when compared with other models. This statistical model may underestimate the need for a hysterectomy, potentially failing to identify patients who actually require the procedure.

To evaluate the performance improvements of our proposed models over the baseline three-path Cascadenet, we conducted statistical testing, with *p*-values provided in Table 3. Our ensemble model showed notable improvement. Although the *p*-value does not meet the conventional threshold for statistical significance (e.g., *p* < 0.05), the ensemble outperformed individual model variations. This finding suggests that the dataset of 60 samples may be too small to detect significant differences. Our individual models did not exhibit differences in performance, and the ensemble method leveraged complementary model strengths, indicating a potential for enhanced robustness and generalization across varied test conditions. Although our proposed method achieved superior performance metrics (90% accuracy, AUC, sensitivity, and specificity compared with baseline), the statistical test suggests that more data might be needed to establish the statistical significance of these improvements.

### 3.2 Ablation Study

To evaluate the contribution of individual models within our proposed ensemble method, we conducted a comprehensive ablation study by systematically removing one model at a time from the ensemble while maintaining the averaging strategy. Table 4 presents the quantitative performance metrics and statistical analysis of each ablated version compared with the complete ensemble. The results demonstrate that removing model 1 or 3 had similar impacts on performance, with accuracy decreasing to 85.0% and AUC dropping to 81.2%. The removal of model 2 or 5 showed moderate performance changes with accuracy at 86.7% and AUC at 85.0%. Interestingly, the ensemble showed robust performance even with the removal of model 4, maintaining an accuracy of 88.3% and achieving an AUC of 90.0%. The *p*-values across all ablations suggest that although each model contributes to the ensemble’s overall performance, the ensemble architecture maintains its robustness without critical dependence on any single model. These findings support our ensemble design choice, demonstrating that the combined approach effectively leverages the complementary strengths of different models while maintaining resilience to individual model variations.

## 4 Discussion and Conclusion

In this study, we developed five machine learning models based on MR images and combined them to predict hysterectomy in pregnant patients with increased risk of PAS using the topographic maps and radiomic features. The performance of the four versions of CascadeNet used on the multimodal data (MRI volumes, radiomic features, and custom feature maps) was significantly improved through architectural changes and fine-tuning, including vision transformers to extract meaningful insights from the custom feature maps. The statistical machine learning model (LightGBM classifier) using radiomics features alone achieved an accuracy of 83.3% (50/60 correct), with an AUC of 0.775. With the ensemble of the five models, we improved the AUC to 0.90 with an accuracy of 90.0%.

The patient populations involved were unbalanced, with the dataset of patients that eventually received a hysterectomy having fewer patients. This imbalance was addressed using techniques, such as stratified cross-validation and usage of class weights in loss function to improve model performance. Finally, we had done extensive testing and optimization to improve our networks, but due to the extremely limited size of our dataset, our test set only contained 60 patients. Our models would consistently get four cases/patients in our test set incorrect with high confidence in its predictions. As such, our models are incapable of correctly classifying those cases, and whatever they may represent, which shows that our training data are not diverse at this time. This could present problems for generalization. However, we have improved upon our previous work, increasing the accuracy by 7%, which shows that there is potential in our methodology and work.

Our approach incorporates the insights gained from Dormer et al.,^44^ where adding topographic feature maps and radiomic features to 3D MRI data slightly enhanced model performance. Specifically, Dormer et al. demonstrated that the addition of each feature type yielded modest improvements in accuracy and AUC, with the highest metrics achieved when all features were combined. This prior research substantiates the predictive value of topographic and radiomic features, justifying their inclusion. Building upon their established feature combination framework, our work focuses on architectural innovations to better leverage these complementary features rather than re-validating their individual contributions. This approach allows us to concentrate on improving the model’s ability to synthesize these proven feature sets more effectively.

Although the radiomic features are extracted from the images segmented by radiologists, which ensures precise identification of uterine and placental regions, this process can be directly applied to the images segmented by deep learning–based segmentation methods, as we previously published.^38,39^ Semi-automated or fully automated segmentation methods, leveraging recent advances in deep learning–based models, have shown promise in the image segmentation tasks and could be trained to identify uterine and placental regions with sufficient accuracy. Moreover, the deployment of automated methods for feature extraction may enhance scalability, allowing for more feasible integration into clinical workflows. Future work could focus on the development of such automated pipelines, which would facilitate the wider application of our predictive model in both large-scale research studies and clinical environments. As deep learning models for medical imaging continue to improve, we anticipate that automated segmentation could minimize radiologist intervention, making our approach more viable for routine clinical use.

We developed models 3 and 5 by employing stratified sevenfold cross-validation as a model selection technique rather than for performance estimation. This approach allowed us to train seven different versions of our modified CascadeNet architecture and modified CascadeNet architecture with ViT, each evaluated on its respective validation data. Although this methodology enabled us to select the model with the best generalization potential, we acknowledge certain limitations. As each fold’s model was evaluated on different validation subsets, we cannot provide traditional cross-validation statistics such as performance standard deviations across folds. Our final performance metrics come from evaluating the selected best-performing model on a completely independent test set, which provides an unbiased assessment of model performance on unseen data. This approach prioritizes selecting the most robust model for clinical application, although it differs from traditional cross-validation approaches. Future work could explore ensemble methods or alternative validation strategies that might provide additional insights into model robustness while maintaining the benefits of our current approach.

Overall, our study highlights the potential of machine learning to predict hysterectomy in pregnant patients with prenatal concern for PAS. By combining models, the accuracy, sensitivity, specificity, and AUC improved. However, our work was not without limitations. Future work will explore using only 2D feature maps for training and prediction, expanding the patient population to improve the model’s robustness. Combining convolutions and transformers with cross-attention mechanisms for processing all the modalities of data might improve the model’s generalization. However, that would be a potential avenue for further exploration. Overall, this research demonstrates the potential of machine learning to pre-emptively predict hysterectomies during pregnancy in patients with PAS, which could have a life-saving impact and pave the way for further development in this field.

## Figures and Tables

**Table 1:** Quantitative analysis of ML models using radiomic features.

Network architecture	Accuracy (%)	Sensitivity (%)	Specificity (%)	AUC

LightGBM	83.3	60.0	95.0	77.5
LogisticRegression	70.0	70.0	70.0	70.0
LogisticRegressionCV	71.6	70	72.5	71.2
PassiveAggressiveClassifier	45.0	55.0	40.0	47.5
Perceptron	66.6	0.0	100.0	50.0
KNeighborsClassifier	46.6	55.0	42.5	48.7
SVC	71.6	55.0	80.0	67.5
MLPClassifier	56.6	50.0	60.0	55.0
DecisionTreeClassifier	73.3	70.0	75.0	72.5
XGBClassifier	76.6	55.0	87.5	71.2
AdaBoostClassifier	73.3	60.0	80.0	70.0
SGDClassifier	66.6	0.0	100.0	50.0
RandomForestClassifier	75.0	55.0	85.0	70.0
GradientBoostingClassifier	76.6	65.0	82.5	73.7
ExtraTreeClassifier	61.6	60.0	62.5	61.2
RidgeClassifier	71.6	70	72.5	71.2

AUC, area under the curve; SVC, support vector classifier; MLP, multilayer perceptron; LGBM, light gradient boosting machine; SGD, stochastic gradient descent.

**Table 2:** Testing results of the three-path CascadeNet method.

Network architecture	Accuracy (%)	Sensitivity (%)	Specificity (%)	AUC

CascadeNet	83.3	85.0	82.5	87.8
Model 1—LightGBM	83.3	60.0	95.0	77.5
Model 2—modified CascadeNet	85.0	90.0	82.5	86.2
Model 3—modified CascadeNet with CV	88.3	85.0	90.0	87.5
Model 4—modified CascadeNet with ViT	83.3	80.0	85.0	82.5
Model 5—modified CascadeNet with CV and ViT	85.0	70.0	92.5	81.2
Ensemble (average)	90.0	90.0	90.0	90.0

**Table 3:** Statistical testing to evaluate the significance of the differences in performance metrics between three-path CascadeNet and each of our proposed models.

Network architecture	*p*-Value

Model 1—LightGBM	0.7518
Model 2—modified CascadeNet	1.0000
Model 3—modified CascadeNet with CV	0.2482
Model 4—modified CascadeNet with ViT	0.4795
Model 5—modified CascadeNet with CV and ViT	1.0000
Ensemble (average)	0.1336

**Table 4:** Impact of component models on ensemble performance in predicting hysterectomy with statistical comparison.

Ensemble	Accuracy (%)	Sensitivity (%)	Specificity (%)	AUC	*p*-Value

Ensemble without model 1	85.0	70.0	92.5	81.2	0.3
Ensemble without model 2	86.7	80.0	90.0	85.0	0.4
Ensemble without model 3	85.0	70.0	92.5	81.2	0.3
Ensemble without model 4	88.3	95.0	85.0	90.0	1.0
Ensemble without model 5	86.7	80.0	90.0	85.0	0.4

## Data Availability

Code and data underlying the results presented in this paper are not publicly available at this time but may be obtained from the authors upon reasonable request.
